# Position Affects Performance in Multiple-Object Tracking in Rugby Union Players

**DOI:** 10.3389/fpsyg.2017.01494

**Published:** 2017-09-08

**Authors:** Andrés Martín, Ana M. Sfer, Marcela A. D'Urso Villar, José F. Barraza

**Affiliations:** ^1^Instituto de Investigación en Luz, Ambiente y Visión, CONICET - Universidad Nacional de Tucumán (UNT) San Miguel de Tucumán, Argentina; ^2^Departamento de Ciencias Básicas, Universidad Tecnológica Nacional San Miguel de Tucumán, Argentina; ^3^Facultad de Ciencias Exactas y Tecnología, Universidad Nacional de Tucumán San Miguel de Tucumán, Argentina; ^4^Facultad de Medicina, Universidad Nacional de Tucumán San Miguel de Tucumán, Argentina

**Keywords:** multiple-object tracking, visual attention, rugby union, perception in sport, performance evaluation

## Abstract

We report an experiment that examines the performance of rugby union players and a control group composed of graduate student with no sport experience, in a multiple-object tracking task. It compares the ability of 86 high level rugby union players grouped as Backs and Forwards and the control group, to track a subset of randomly moving targets amongst the same number of distractors. Several difficulties were included in the experimental design in order to evaluate possible interactions between the relevant variables. Results show that the performance of the Backs is better than that of the other groups, but the occurrence of interactions precludes an isolated groups analysis. We interpret the results within the framework of visual attention and discuss both, the implications of our results and the practical consequences.

## Introduction

Skilled perception is an important determinant of performance in sports that are characterized by a complex and rapidly changing environment (Starkes, 1987; Helsen and Starkes, 1999; Williams et al., 1999; Ward and Williams, 2003). In rugby union, for example, players must combine the information picked up from the ball, team-mates, and opponents in order to make the appropriate decisions according to their objectives in the game. However, not all the information present in the visual field is relevant to those goals. For this reason, the process of collecting information needs to separate relevant information from noise. The mechanism that accomplishes this selection is called visual attention (James, 1890; Posner, 1980; Duncan, 1984) and represents a key factor underlying perceptual skill in sport (Abernethy, 1988; Williams, 2000; Ward and Williams, 2003; for a review, see Moran, 2010). Note that this definition refers to a specific meaning of the term attention, namely selective attention (Carrasco, 2011). However, the term “visual attention” embraces a variety of concepts (Harris and Jenkin, 2001). In fact, the existence of different types or sub-processes of attention (Coull, 1998) such as selective attention, attentional orientation, divided attention, and sustained attention has been suggested.

A number of investigations addressed these attentional issues in an attempt of understanding expertise in sport (for a review, see Memmert, 2009). Researchers took advantage of all available methodological tools developed during the last years to quantify attention and its effects on tasks performance. For example, Lum et al. (2002) used a cueing paradigm to show that soccer and volleyball players were better at voluntarily orienting attention to locations where useful information was most likely to occur. More recently, Hüttermann et al. (2014) studied how athletes of different sports distribute their attention in comparison to novices. Results show that experts have a greater attention breadth than novices in a dimension that depends on the specificities of each sport. Currently, based on the idea of “quiet eye period” (Vickers, 2007), much efforts are being devoted to assess attention in experts athletes through techniques of pupilometry (for example, Unsworth and Robison, 2015; Moran et al., 2016).

These studies strengthen the intuition that an expert player may learn to focus attention on spots of interest, and that this particular skill could show up as an enhanced performance relative to non-players in those tasks embedded within paradigms designed to test attention.

However, as often occurs in science, not all the empirical evidence is in agreement. For example, studies performed by using the Multiple-Object-Tracking paradigm (MOT) showed that air-traffic controllers perform much better in this task than a group of undergraduate students (Allen et al., 2004). More recently, Faubert (2013) showed that professional athletes are much better than sub-elites and novices at learning complex and neutral dynamic visual scenes. Conversely, Memmert et al. (2009) showed that the expected differences in performance between expert and novice handball players did not appeared in a similar MOT task. This failure to find the expected effect leads to question the methods and the reliability of the quantities obtained at the end of our experimental procedures. On this ground, we aim, on one hand, to add evidence about the attentional capacities acquired by the sport practice and, on the other hand, to explore some methodologies of data analysis and experimental designs that could help us show, in case they should exist, differences between the evaluated groups.

Our rationale can be summarized as follows. Cavanagh and Alvarez (2005) argue that many tasks, (such as those performed in some team sports, video games, and military activities) require participants to track multiple targets simultaneously so one might expect them to have more developed attentional skills. As an example, the authors mention that “the icehockey champion, Wayne Gretzky, for example, was said to keep track of all the players on his and the opponents' team” (Cavanagh and Alvarez, 2005, p. 349), alluding to his enhanced divided attention. These differences between experts and novices should be measurable. One possibility to test it is the multiple-object tracking paradigm (MOT; Pylyshyn and Storm, 1988) which was suggested to be suitable for testing divided attention (Intriligator and Cavanagh, 2001; Green and Bavelier, 2003, 2007; Allen et al., 2004 for a review, see Green and Bavelier, 2012). Previous studies have failed in finding any difference between experts athletes and novices by using MOT (Memmert et al., 2009). However, the authors performed the experiment in a specific condition: they measured accuracy for the maximum speed at which the observers could track the items. This maximum speed is normally relatively low (in fact, when using this speed, accuracy reaches levels of around 90%). Hence, it is reasonable to expect good performances from all the participants. On the other hand, if the conditions were extremely difficult, we could expect that performance would be around chance for all the participants. Between these two situations, there should be a range in which the task is more demanding but still performable. Thus, if the goal is to discriminate an alleged improved ability, one should test such situations. In order to do this, other variables, such as stimulus duration and speed are included in our experimental design.

## Materials and methods

### Procedure

Before conducting the main experiment in which we measured accuracy in the MOT task, we performed a preparatory session to estimate the top tracking speed for a stimulus configuration consisting of 8 items (4 targets and 4 distractors) presented during 6 s. In this session, observers adjusted the speed of the circles by pressing the arrow keys (left arrow to slow down, right arrow to speed up). Observers were instructed to increase the speed until they found that they were moving too fast to track. Each trial began with a speed that was the mean between the initial speed of the preceding trial and the speed limit obtained in that trial. This procedure was repeated 10 times until the observers reached the maximum speed at which they could track all of the targets for about 6 s. Ten observers participated in this speed session. We determined the speed limit by averaging the results of these 10 observers and it was used to determine the range of conditions for the main experiment. Table 1 shows the average speed limit obtained in the preparatory session. This value was used as a reference for setting the range of speed in the accuracy (main) experiment.

**Table 1:** Maximum Speed limit computed upon 10 non-athletes observers with a fixed presentation time of 6 s.

**Mean**	**Std. Dev**	***n***
16.33	2.041	10

*We assume that this Speed and Time represent the easiest condition in the MOT task. Note that the Speed variable in the main experiment was set to 16.8, 21.2, and 25.5 deg/s and the Time variable was set to 6 and 12 s*.

**Figure 1:**
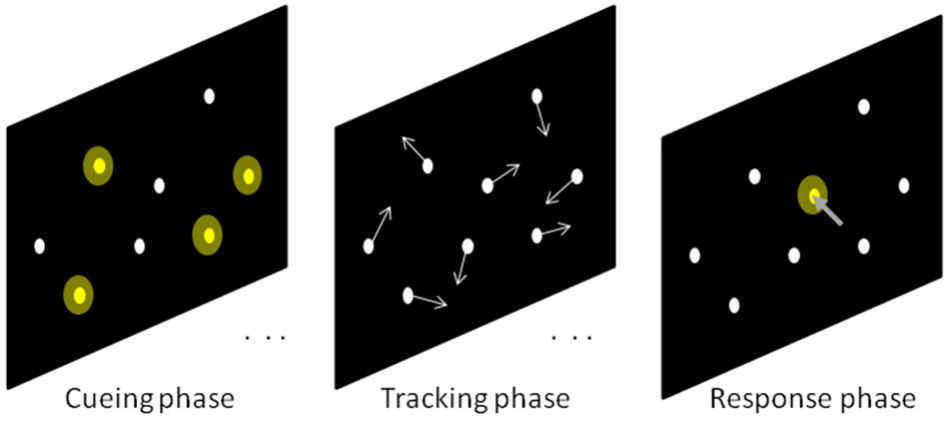
Illustration of the sequential phases included in a trial of the main experiment. Cueing phase: eight static dots were displayed on the monitor. The four targets (shaded in yellow) were signaled by making them to blink during 4 s. Tracking phase: The blinking stopped and all dots started moving with identical speed along random trajectories. Response phase: Once the tracking phase finished, the dots were frozen and the observers had to click with the mouse over the targets. Whenever a click was correct, that dot changed its color to inform the correct response.

The main experiment all the participants performed each experimental condition 20 times (trials). The order in which the observers performed the six different conditions was randomized to balance any effect of learning. Notwithstanding we took this precaution in the experimental design, we recorded the trial number in order to incorporate this possible fixed effect source to the model. Each trial began with the cueing phase which was followed by the tracking phase. At the end of this phase, the circles stopped and the observers had to point and click with the mouse over the tracked targets (response phase). Each time the observer clicked over a target circle (correct response), that circle changed its color giving a feedback signal to the observer. To codify performance we counted the number of correct responses and defined it as “good” if the correct responses were 3 or 4 (higher than chance). In the next section we explain in detail all the data analysis. Figure 1 illustrates the three phases of the stimulus presentation.

### Stimuli and apparatus

Stimuli consisted of eight white circles (1° in diameter) presented on a black background (39.7° × 23.4°). The initial positions of the circles on the screen were chosen at random. Four of them were targets and were cued by flicking before the beginning of each trial (cueing phase). The other four circles were distractors. Immediately after the cueing phase, the circles began to move at a constant speed over random trajectories. When a circle reached the edge of the screen, it changed its direction, as if it were an optical reflection. There were no additional constraints in the circles' trajectories so there was the possibility that they occluded one another for an instant. The speed and the duration of the stimulus were the independent variables of this experiment (three speeds × two durations = six conditions). The speeds were 16.8, 21.2, and 25.5 deg/s (8, 10, and 12 pixels/s, respectively), and the durations 6 and 12 s. The speed values were chosen in order to generate a range of conditions, from low (low speed and short stimulus duration) to high (high speed and long stimulus duration) demand. The least demanding condition was defined based on a preparatory experiment that will be explained in the next section.

Observers performed the experiment supporting their chin and forehead on a chinrest located 0.60 m from the screen. The experiment was run on a PC equipped with a high-performance video card, by using MATLAB and Psychophysics Toolbox V3 (Brainard, 1997; Pelli, 1997).

### Participants

Eighty-six rugby union players aged 16 to 35 (mean = 18.7, SD = 3.5), belonging to different teams of the province of Tucumán, Argentina, participated in this experiment, in addition to a control group made up of 16 undergraduate students aged 18 to 25 (mean = 21.6, SD = 2.5) with no systematic experience in sport. Therefore, a total of 102 participants take part in this experiment.

The distribution of players according to their ages is as follows: 58 players aged 16 to 18 and 34 players over 18. The distribution of players according to their positions is: 7 Hookers, 13 Props, 9 Locks, 14 Flankers, 2 Number eights, 7 Fly-halfs, 11 Scrum-halfs, 10 Centers, 8 Wings, and 5 Full-backs. This corresponds to 45 Forwards (Hooker, Prop, Lock, Flanker, and Number eight) and 41 Backs (Full-back, Wing, Fly-half, Scrum-half, and Center).

The process of selection of the participants was as follows: we summoned the coaches of all the Tucumán teams and asked them to invite all their players over 15 years old, to participate voluntarily in our study. We explained the goal of the study to the coaches and clarified that the players had to remain naive as to the purpose of the investigation. There was no additional criterion for the selection of the players so that all the players who came to the laboratory were welcome. The protocol was approved by CEI-UNT (Ethic Committee for Research of the National University of Tucumán, Argentina, Resolution: 1466/16), and followed all the procedures in order to protect the privacy and security of the participants, according to the tenets of the Declaration of Helsinki. All participants signed an informed consent.

All participants had normal or corrected-to-normal vision.

### Data analysis

The observed variables were: Performance (dependent variable), which refers to the behavior of each observer in each trial, and was classified as Good (code 1) if the observer correctly choose 3 or 4 circles and Bad (code 0) if otherwise (a Bernoulli variable); Speed (independent variable), which is the circles' speed in each test, a factor with three levels (Slow = 16.8 deg/s, Medium = 21.2 deg/s and Fast = 25.5 deg/s); Time (independent variable), which refers to the length the circles kept moving on the screen, a factor with two levels (Short = 6 s and Long = 12 s); Position (independent variable), which refers to the position of the observer on the field and the control group, a three level factor (Control, Backs and Forwards); and Repetition (independent variable), that codes the order of the tests, ranges from 1 to 120 (20 ^*^ 6, six conditions of Speed and Time by 20 repetitions each).

As a result, we have a study of repeated measures with binary responses. Therefore, we choose a Generalized Linear Mixed Model (GLMM) as a suitable model to fit our data. The adjustment was made through the software R Core Team (2017) with the package lme4 (Bates et al., 2015) with Binomial family and logit link function.

The model assumes that the performance observed is a random variable that can be represented as:

$$\begin{array}{l} Y_{ij}with\;i=1,2,\ldots ,102\wedge j=1,2,\ldots ,120 \end{array}$$

Where the subscripts i and j represent observer and repetition, respectively. The observations of the response, given the random effect associated with the subject (*Y*_*ij*_/*b*_*i*_) are independent and follow a Bernoulli distribution with parameter π_*ij*_, i.e.:

$$\begin{array}{l} \forall i=1,2,\ldots ,102Y_{ij}/b_{i}\beta \left(\pi_{ij}\right) \end{array}$$

Then, the link function to consider is: $g\left(x\right)=\text{ln}\frac{x}{1-x}$. So the model is:

$$\begin{array}{l} \forall i=1,2,\ldots ,102g\left[E\left(Y_{ij}/b_{i}\right)\right]=ln\frac{\pi_{ij}}{1-\pi_{ij}}=X_{j}\beta +Z_{i}b_{i} \end{array}$$

With $b_{i}\sim N\left(0,\sigma_{i}^{2}\right)$. *Y*_*i*_ is a vector 120 × 1, *X* is the design matrix of fixed effects 120 × 13, β is the vector of fixed effects coefficients and interactions 13 × 1; *Z* is the design vector of random effects 102 × 1.

In summary, the model, in addition of observing the experimental design, aims to capture the probability^1^ of a Good response (i.e., the probability of a success in the selection of more than two circles out of four) conditional to Speed and Time (by which we have set the difficulty of the task or which jointly set the difficulty of the task) and Group (which refers to the kind of observer doing the task).

## Results

**Figure 2:**
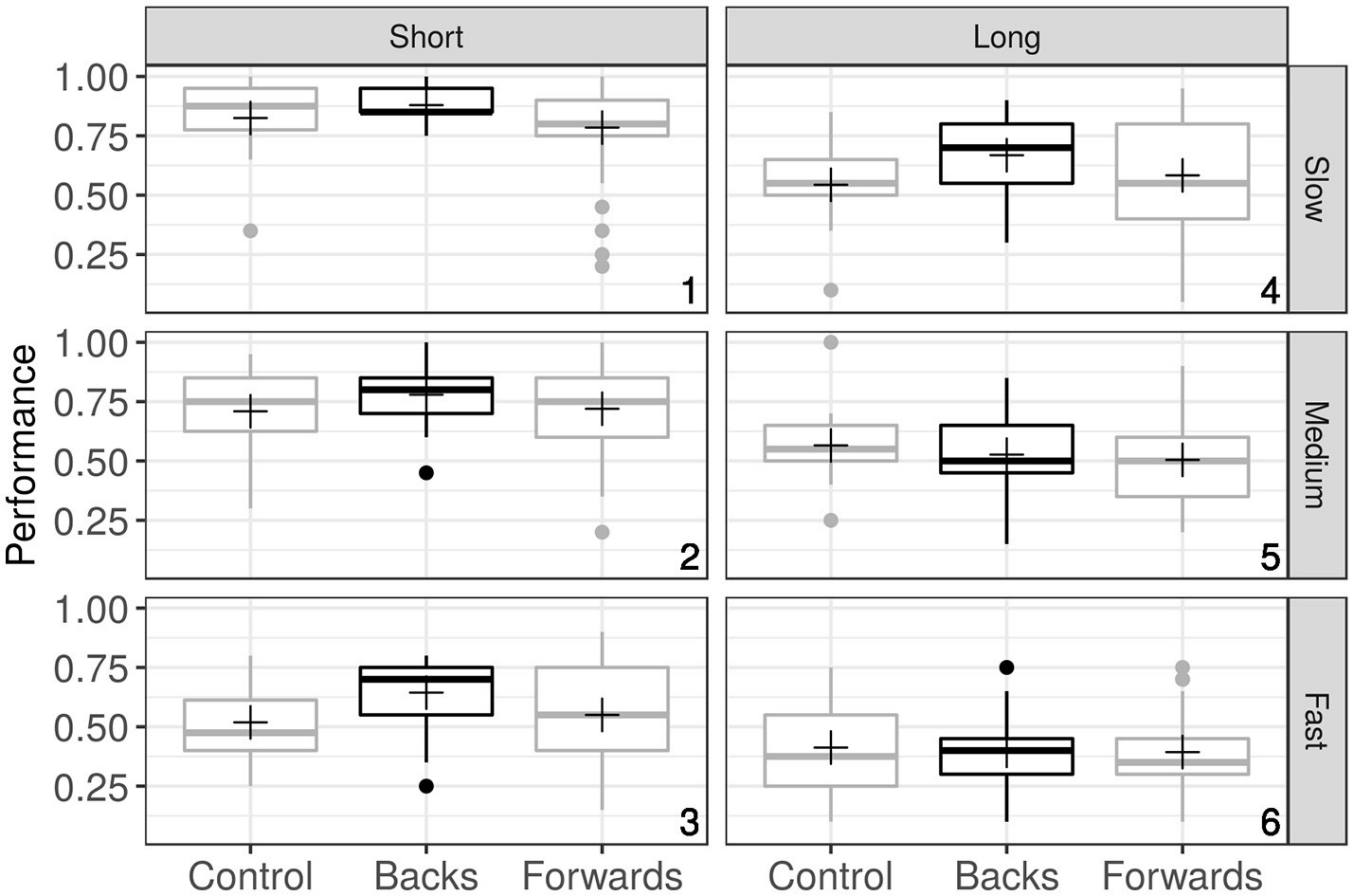
Box-plots summarizing the data grouped over Speed (rows), Time (columns) and Observer's group (X axis). Each plot represents Performance (the probability of a Good response) for the three groups of observers. Crosses indicates mean values, and the numbers in the right bottom corners of the plots represent the particular combination of Speed and Time that codifies the task difficulty (Condition).

Figure 2 shows the box-plots summarizing the proportion of performances Good for the six experimental conditions and the three groups of observers. The crosses represent the mean performance for each group, and the numbers at the lower right corner in the panels refers to the difficulty of the task as a particular combination of Speed and Time (see the caption).

The plots clearly show that the proportion of Goods (i.e., hitting at least three circles out of four in the MOT task) depends on the particular combination of speed and time: higher speeds and longer times increase the task difficulty, which results in a decrease of Good responses. This is a well-known effect in the MOT tasks (Liu et al., 2005; Mitroff and Alvarez, 2007; Franconeri et al., 2010). In those panels in which the difficulty of the task was too easy (panel numbered 1) or too hard (panel numbered 6), there is an homogeneous behavior of the groups and there is no discernible differences among them.

However, in panels 2, 3, and 4, in which the difficulty is intermediate, the Backs group has a better performance than the others. In contrast, there is no instance in which a (obvious) better performance of the Forwards or the Control group are found. Is this observed pattern of responses a manifestation of a group effect or merely an incidental observation due to randomness? The proposed model, depicted in the preceding sections, will help us to shed light on this question.

Table 2 summarizes the result of fitting the model by using the GLMM. The random effects are shown in the upper part of the table, and the lower part shows the fixed effects. The Std Dev of the random effects represents the variability of the mean response for each observer (mean response across repetitions of the task) respect to the intercept. Note that these random intercepts do not have base or reference levels. They are increments or decrements to the overall intercept for each observer. The random term was included in the model because our sample represents a much bigger universe of observers. Therefore, what matters is to know whether the random effect affects similarly the three groups considered in our sample. The caterpillar plots shows that each group of observers displays a similar distribution, which are, in turn, similar to the global distribution (please, see the Supplementary Material).

**Table 2:** Model summary.

**Group**	**Name**	**Variance**	**Std. Dev**.	
**RANDOM EFFECTS**				
Observer	(Intercept)	0.33	0.58	
**FIXED EFFECTS**				
	**Estimate**	**Std. Err.**	**Z-value**	**P-value**
(intercept)	1.168	0.181	6.455	0.000
SpeedMedium	−0.363	0.127	−2.859	0.004
SpeedFast	−1.116	0.125	−8.946	0.000
TimeLong	−0.845	0.101	−8.366	0.000
GroupBacks	0.630	0.215	2.932	0.003
GroupForwards	−0.004	0.210	−0.017	0.986
Rep2	0.003	0.001	5.535	0.000
SpeedMedium:GroupBacks	−0.371	0.150	−2.471	0.013
SpeedFast:GroupBacks	−0.198	0.148	−1.337	0.181
SpeedMedium:GroupForwards	−0.040	0.146	−0.270	0.787
SpeedFast:GroupForwards	0.057	0.144	0.396	0.692
TimeLong:GroupBacks	−0.334	0.121	−2.765	0.006
TimeLong:GroupForwards	0.000	0.000	−627.000	0.000

*Top: random effects; as the model has only a random intercept term, there is only one reference to its variance, see the text for details. Bottom: fixed effects and interactions coefficients; First Column: (Intercept) refers to the basal condition (SpeedSlow, TimeShort, GroupControl); SpeedMedium and Speed Fast are levels of the factor Speed; TimeLong is a level of the factor Time; GroupBacks and GroupFordwards are levels of the factor Group; Rep2 codes the repeated measures we took for each observer, from the first to the 120th trial; the remaining terms corresponds to the interactions. Estimate column: estimated coefficients values in log odds units; the remaining columns are the standard error, the Z-value and the p-value associate with each estimation, see the text for commentaries*.

The first column of the fixed effects contains the estimates of the model's coefficients in terms of the link function (i.e., the coefficients for the fixed effects are log odds of success). The fourth column shows the level of significance of the coefficients. These values are approximate, and were calculated by using the Wald test (Fears et al., 1996; Pawitan, 2000). The two central columns (Std Error and *z*-value) were shaded to facilitate the reading of the table.

The term “intercept” encodes the reference level, which was arbitrarily chosen as GroupControl, TimeShort, and SpeedSlow. In terms of our experimental design, this reference level corresponds to the easiest MOT condition (numbered 1 in Figure 2). All the coefficients must be interpreted in relation to this term. For example, the performance decrement due to changing from SpeedSlow to SpeedFast implies a reduction in the log odds of the order of the intercept: that is, from almost perfect to near chance. This is what it can be appreciated in the box-plots of Figure 2 if one compares the Panels 1 and 3 for the Control group. We cannot ignore that there are interactions between Group and both, Time and Speed. This situation precludes an isolated examination of the fixed effects.

Consequently, the model indicates that the strongest effects correspond to speed and time, which was clearly expectable according to the data present in the literature (Liu et al., 2005; Mitroff and Alvarez, 2007; Franconeri et al., 2010). Moreover, the model shows that there is a significative effect of the group Backs, and that this effect increases the log odds, which in turn means that an observer belonging to this group is more likely to give a better performance, although we have to take into account the interactions with the other variables. In contrast, the group Forwards does not show any significative effect and thus, it is indistinguishable from the control group. Finally, the model shows that there is a small but significative effect of the variable Repetition (Rep2), which suggests a certain effect of learning (from the first to the 120th trial).

**Figure 3:**
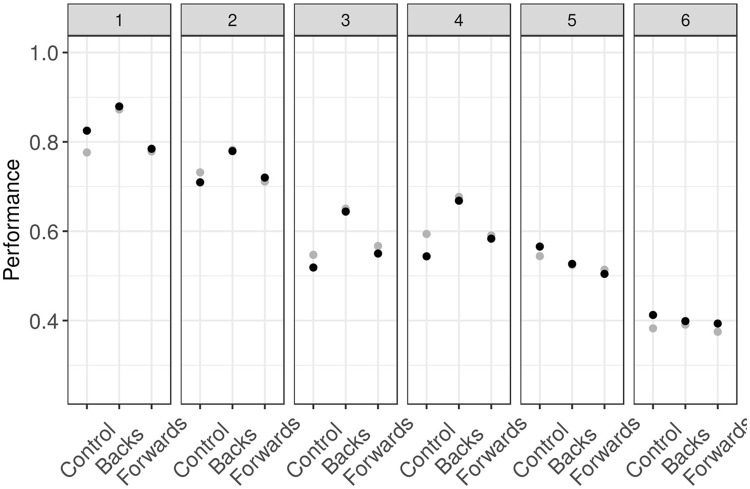
Model predictions (gray dots) against observed performance (black dots) across conditions (columns). The figure shows the agreement between the model and the data.

Figure 3 shows a comparison between the model mean predicted values and the mean performance observed for each group and condition. The plot clearly shows a good concordance between these two groups of data (the model prediction is in gray and the experimental data are in black), which indicates that the model captures the mean general behavior of the observers across the different conditions.

## Discussion

In this study, we have measured the performance of 86 rugby union players and a control group in a MOT task to explore whether we could find differences between players and non-players and between players that occupy different positions in the field. To achieve this goal we performed a thorough statistical analysis by using GLMM. Both, descriptive and statistical analysis, show overall differences between the Backs and the other two groups (Forwards and Control), although, as we mentioned before, these differences do not appear, or are very small, in some specific experimental conditions.

A visual inspection of the box-plots of Figure 2 suggest that, in general, the group Backs performs better than the other groups. This suggestion is quantified by fitting the model to the data. This quantification indicates that the probability to succeed in the MOT task of an observer belonging to whichever group is conditional to both Speed and Time (i.e., there are interactions). While we cannot ignore the presence of interactions, it is also important to note that the model coefficients signals a significative effect of the Backs upon performance and a small (but also significative) effect of learning (coded as repetition in our experimental design). Any interpretation of these results must consider that the interactions reflects a complex scenario in which, defining a standard stimuli or experimental design for a MOT experiment with the aim of studying particular populations, is not trivial. Even less if one wants to draw conclusions about differential behaviors among groups. In contrast, what these interactions suggest is that any experiment must cover an appropriate range for the critical variables and the conclusions must be based on the whole set of data.

We interpret the results of performance of the three groups in terms of the previous discussion. We found a significant effect of the group Backs on performance that is conditional to Speed and Time. It is accepted that the MOT task puts at stake the mechanisms of divided attention (Intriligator and Cavanagh, 2001; Green and Bavelier, 2003, 2007; Allen et al., 2004 for a review, see Green and Bavelier, 2012). Differences in performance in this task may be attributed to differences in attentional factors. The three groups of participants of this study present particularities that could affect their attentional capacities differentially. Therefore, we hypothesize that the difference in performance found among groups could reflect the effect of an augmented attentional capacity of the Backs respect to the Forwards and Control. Such particularities refers first to the fact that participants belonging to the groups Backs and Forwards systematically practice sport (Rugby Union). Second, Backs and Forwards are engaged in very different roles in the game. Third, Backs and Forwards, typically, perform different training programs in the team.

Interestingly, we are not the first in suggesting the existence of differences in the attentional capacities of different groups of rugby union players. Based on the Test of Attentional and Interpersonal Style (TAIS; Nideffer, 1976), Maynard and Howe (1989) and Di Corrado et al. (2014) found that the attentional capabilities of rugby union players are affected by age, experience and position. TAIS consists in defining an attentional style that is capable of identifying a group of athletes, based on athlete's self-perception about its own strategies. In particular, the results of Maynard and Howe (1989) suggest that Halfbacks have a superior attention span to process multiple information sources, integrate them and make good decisions.

But, how confident can we be that this is the best explanation for our results? Our hypothesis assumes a transfer of learning from specific activities of the game, to the MOT task. However, this interpretation may be challenged by some studies about cognitive training. Boot and collaborators (Boot et al., 2011, 2013) for example, analyzed in detail the case of transferring learning from action video games to the MOT task, and suggested that the alleged properties of the video games could actually be the result of a placebo effect (among others) rather than a real transfer of training. We believe that our experiment can be considered free of this effect for two reasons. First, because the players who participated were not recruited as experts to be compared to novices, thus, there would not be a special motivation in these players. Furthermore, there is no reason to suppose that athletes have, themselves, special motivations toward this type of laboratory tasks that might otherwise be attributed to the undergraduate students of the control group. Finally, we can assume that there are really no different motivations between backs and forwards with respect to this task.

Another possibility would be that our results were reflecting some bias in the sample of players that participated in the experiment. In this respect, we think this possibility can be ruled out by the fact that the participants' recruitment was performed by rugby union coaches, following our protocol, which welcomed all applicants. In addition, we have included in the analysis all the participants.

Finally, we have to question ourselves what are the implications of these results. If one accepts that Backs are better in the MOT task because they develop in their sport practice better attentional capacities, then we may suggest that it is likely that these capacities are useful for that sport practice. Then, how disadvantageous can it be for a player the lacking of these augmented capacities? Is it possible to train these skills and reflect on sports performance? How can be measured the impact of these workouts on team performance? These are questions that exceed the scope of this study but, necessarily have to be addressed if one wants to transfer this knowledge to the game field.

## Ethics statement

All subjects gave written informed consent in accordance with the Declaration of Helsinki. We followed the protocols and guidelines of CONICET (National Research Council of Argentina).

## Author contributions

AM, contributed with the experimental design and data analysis. He also participated in the manuscript writing and final approval of the version to be published. AS contributed with the model formalization and data analysis. She also participated in the final approval of the version to be published. MD contributed with the model formalization and data analysis. She also participated in the final approval of the version to be published. JB contributed with the experimental design, MOT task design and data acquisition. He also participated in the manuscript writing and final approval of the version to be published.

### Conflict of interest statement

The authors declare that the research was conducted in the absence of any commercial or financial relationships that could be construed as a potential conflict of interest.

## Footnotes

^1^Strictly, a GLMM model with a logit link function represent the change in the odds due to changes in the predictor's variables, but with the odds it is possible to compute, easily, the probabilities of a “Good” response and this numbers are more understandable in the current context.
