# Supplementary material for: Position Affects Performance in Multiple-Object Tracking in Rugby Union Players
Source: Front Psychol. 2017 Sep 8;8:1494. doi: 10.3389/fpsyg.2017.01494 (PMC5599788; doi:10.3389/fpsyg.2017.01494)
Supplement: Supplementary file 2 [file DataSheet2.PDF]

Script to analize the Rugby Data

library(ggplot2)

library(dplyr)

library(tidyr)

library(lme4)

1) Read Data (Set the working directory properly)

Data <- read.csv("RawRugbyData.csv")

“RawRugbyData.csv” is a comma separated doccument with a header and 12240 rows of data. The header contains the columns names for each variable, and each row is an observation.

The variables are:

Observer: the initials of the observers

Performance: how many hits per trial (0 = 0; 25 = 1; 50 = 2; 75 = 3; 100 = 4)

Group1: for the player group, indicates their desagregated position in the field

Group2: for the palyer group, indicates their position in the field

Time: how long last the stimulus on the screen (Factor; levels = Short / Long)

Speed: how fast was the motion of the circles in the MOT task (Factor; levels = Slow / Medium / Fast)

Rep1: which number of repetition whithin each condition (From 1 to 20)

Rep2: which number of repetition across conditions (From 1 to 120)

PerfBin: performance coded binomialy: 3 or more hits = 1, else 0

Condic: a combination of Speed and Time (Factor; levels = 1 to 6)

2) Set factor order and rename variables

Data$Group < -factor(Data$Group2, levels = c(“Control”,“Backs”,“Forwards”)) Data$Time < -factor(Data$Time, levels = c(“Short”,“Long”)) Data$Speed < -factor(Data$Speed, levels = c(“Slow”,“Medium”,“Fast”)) Data$BinaryPerf < -Data$PerfBin Data$Condition < -Data$Condic

3) Summarize

SumData <- Data %>% group_by(Observer, Group, Time, Speed, Condition) %>% summarise(ProbPerf = sum(BinaryPerf)/n())

4) Draw the boxplot (Desciptive Statistics)

BoxPlot <- ggplot(data = as.data.frame(SumData), aes(Group, ProbPerf, color = Group)) + geom_boxplot() + theme_bw() + ylab("Performance") + facet_grid(Speed ~ Time) + stat_summary(fun.y=mean, colour="black", geom="point", shape = 3, size = 3) + geom_text(aes(x = 3.5, y = 0.1 , label = Condition), color = "black", size = 4) + theme(legend.position = "none", text = element_text(size = 12), axis.title.x=element_blank(), axis.text.x = element_text(size = 12), axis.text.y = element_text(size = 12)) + scale_colour_manual(values = c("grey70", "black", "grey70"))

5) Fit the model

FullModel <- glmer(BinaryPerf ~ (Speed + Time) * Group + Rep2 + (1|Observer), data = Data, family = binomial, control = glmerControl(optimizer = "bobyqa"))

6) Predictions

Predictions <- predict(FullModel, type = "response") Results <- Data %>% mutate(Preds = Predictions)

7) Results summary

ResumeOut <- Results %>% group_by(Group, Condition) %>% summarise(ProbGood = mean(Preds), ProbPerf = sum(BinaryPerf)/n()) %>% gather(key = Probs, value = Prob1, ProbGood, ProbPerf) %>% data.frame()

8) Draw result chart

PredictionsPlot <- ggplot(data = ResumeOut, aes(Group, Prob1, color = Probs)) + geom_point() + theme_bw() + ylab("Performance") + facet_wrap(~Condition, 1, 6) + theme(legend.position = "none", text = element_text(size = 12), axis.title.x=element_blank(), axis.text.x = element_text(size = 12, angle = 45, vjust = 0.5), axis.text.y = element_text(size = 12)) + scale_colour_manual(values = c("grey70","black")) + ylim(c(0.25,1))

Caterpillar figures

1) extract random effects

RandomsEf <- ranef(FullModel, condVar=TRUE) Randoms.DF <- data.frame(Observer = rownames(RandomsEf[[1]]), int=unname(RandomsEf[[1]]), se=sqrt(c(attr(RandomsEf[[1]],"postVar"))), row.names = NULL)

2) include group factors

GroupedData <- Data %>% group_by(Observer, Group) %>% summarise(ProbPerf = sum(BinaryPerf)/n()) %>% select(Observer, Group) Randoms.DF <- Randoms.DF %>% left_join(GroupedData) %>% arrange(int)

3) Plot the caterpillar

a) All toghether

ggplot(Randoms.DF, aes(reorder(Observer, int), int, ymin=int-1.96*se, ymax=int+1.96*se)) + geom_pointrange(aes(colour= Group)) + coord_flip() + xlab("Observers")

b) faceted by Group

ggplot(Randoms.DF, aes(reorder(Observer, int), int, ymin=int-1.96*se, ymax=int+1.96*se)) + geom_pointrange(aes(colour= Group)) + coord_flip() + facet_grid(Group ~ .) + xlab("Observers")

Save the graphs in a suitable format

ggsave("RugbyBoxPlot.tiff", plot = BoxPlot, width = 15, height = 10, units = "cm")

ggsave("RugbyPredsPlot.tiff", plot = PredictionsPlot, width = 15, height = 10, units = "cm")
