# Supplementary material for: Position Affects Performance in Multiple-Object Tracking in Rugby Union Players
Source: Front Psychol. 2017 Sep 8;8:1494. doi: 10.3389/fpsyg.2017.01494 (PMC5599788; doi:10.3389/fpsyg.2017.01494)
Supplement: Supplementary file 3 [file DataSheet3.DOCX]

Supplementary Material

Assessment of attention in rugby union players using Motion-Object Tracking

Andrés Martín^*^, Ana M. Sfer, Marcela A. D’Urso Villar, José F. Barraza

*** Correspondence: Andrés Martín**: amartin@herrera.unt.edu.ar

# Supplementary Figure Captions

**Supplementary Figure 1.** Caterpillar plot of the whole set of random effects. Colors represent the groups: Red, Control; Blue, Backs; Green, Forwards. There is no any structure that signal a particular behavior for each group.

**Supplementary Figure 2**. Caterpillar plots of the random effects faceted according to the group. As in the previous plot, there is no any structure that signal a particular behavior for each group.
