# Supplementary figures and images for: Position Affects Performance in Multiple-Object Tracking in Rugby Union Players
Source: Front Psychol. 2017 Sep 8;8:1494. doi: 10.3389/fpsyg.2017.01494 (PMC5599788; doi:10.3389/fpsyg.2017.01494)

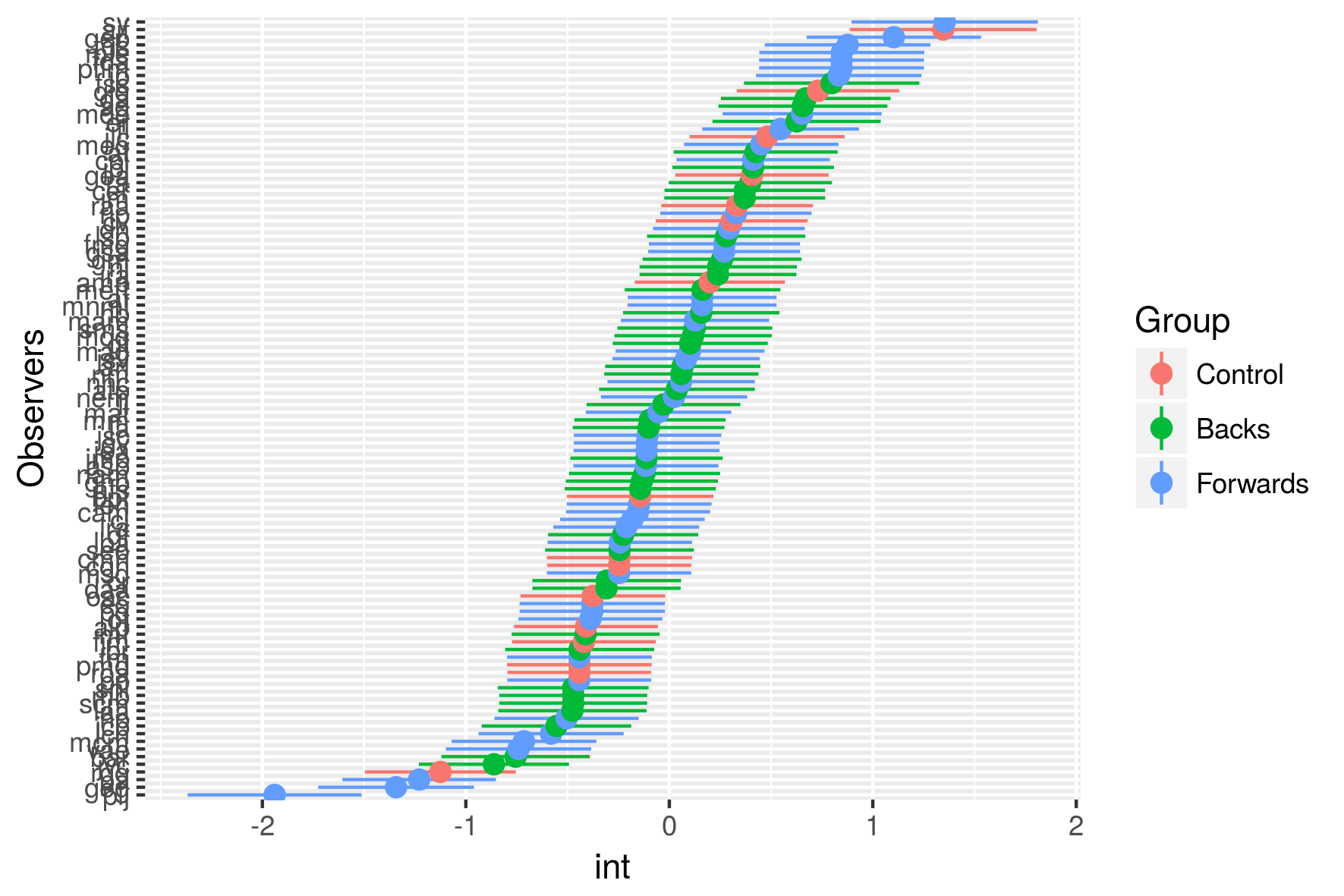

Supplement: Supplementary file 4 [file Image1.TIFF]

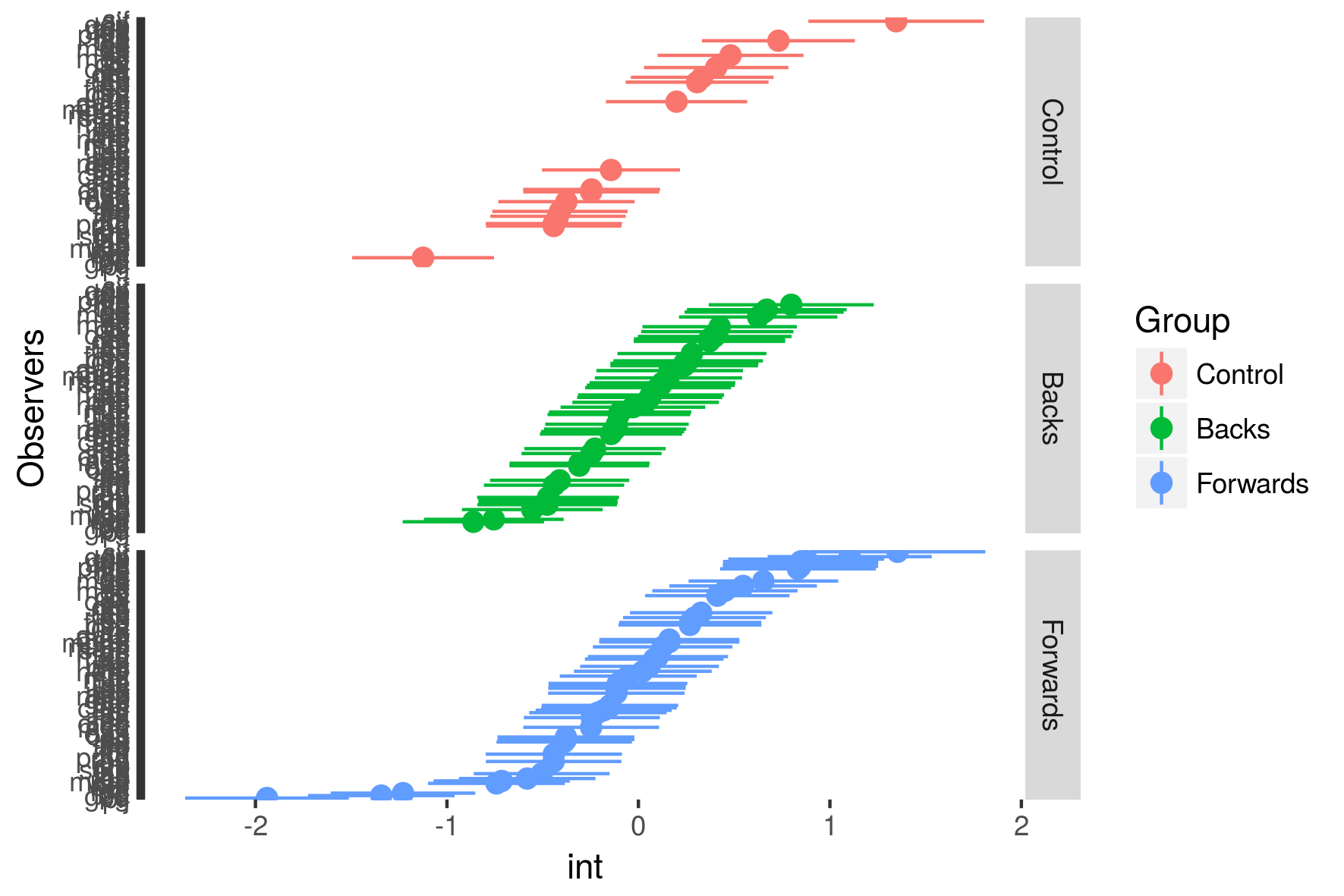

Supplement: Supplementary file 5 [file Image2.TIFF]
